# The commission of crime from the perspective of decision-making differences

**DOI:** 10.3389/fpsyg.2022.937876

**Published:** 2022-10-10

**Authors:** Jiaxi Peng, Jiaxi Zhang, Weizhuo Yuan, Xuan Zhou, Jianquan Tian, Peng Fang

**Affiliations:** ^1^Mental Health Education Center, Chengdu University, Chengdu, China; ^2^Xi’an Research Institute of High-Technology, Xi’an, China; ^3^Naval Medical Center, Shanghai, China; ^4^Department of Military Medical Psychology, Air Force Medical University, Xi’an, China

**Keywords:** criminal decision-making, crime, decision-making differences, relative deprivation, decision-making styles

## Abstract

A criminal act can be regarded as an irrational decision-making process. Therefore, understanding differences in the criminal decision-making process would shed light on criminal behavior. We utilized dual processing theory to propose that offenders’ differences in decision-making may cause them to adopt non-adaptive behaviors, such as high reference point setting, abnormal reward–punishment sensitivity, delayed discounting rate, and decision-making style. Our study compares differences in these indicators between offenders (*n* = 518) and non-offenders (*n* = 636) in a diverse sample of Chinese adults. The results showed that compared with non-offenders, offenders had higher relative deprivation, reward sensitivity, and delayed discounting rates but lower punishment sensitivity and vigilance in decision-making. A logistic regression analysis also shows that the above factors were significant predictive indicators for the commission of crimes.

## Introduction

Economists and cognitive behavior psychologists, along with criminologists, are striving to interpret the decision-making process that underlies criminal activity. Unfortunately, the vast majority of empirical research on criminal decision-making takes the classical school of Cesare Beccaria and Jeremy Bentham as its framework, and models of criminal decision-making have been grounded in the hypothesis that the decision to commit a criminal act originates from the offender’s assessment of the anticipated net utility of the act (Beccaria, 1986/1764; Piliavin et al., 1986; Bentham, 1988/1789; Grgic-Hlaca et al., 2018). However, from the perspective of normative decision-making, commission of a crime represents bad decision-making behavior that is both irrational and cost-ineffective; therefore, some scholars, such as Van Gelder (2013) claimed that offenders make improper decisions because of differences in their decision-making process relative to that of people who do not commit crimes. Cornish and Clarke (1986, 1989) suggested that offenders make decisions to commit crimes based upon adequate or accurate information, displaying limited rather than normative rationality. This study, then, attempts to explain the commission of a criminal act from the perspective of differences in the decision-making cognitive process by comparing Chinese offenders and non-offenders.

### The prospect theory model and the commission of crime

**FIGURE 1:**
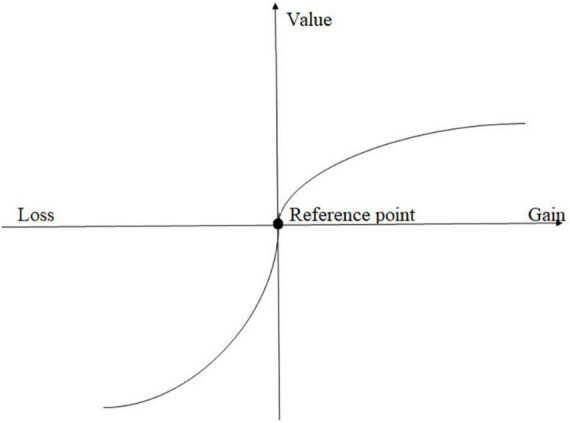
The value function [adopted from Tversky and Kahneman (1992)].

The early criminal classical school argues that crime is a result of a hedonistic calculus, and it was a choice attributed to differences in costs and benefits from the criminal activity (Beccaria, 1986/1764; Bentham, 1988/1789). With the development of behavioral decision-making research, however, more mature theoretical models have been introduced (Van Gelder and De Vries, 2014). For instance, prospect theory holds that the choice of behavior is based on judgment of prospects. This theory is quantified with a mathematical expression, V = Σπ(p)v(x) (Tversky and Kahneman, 1992). The presence of the value function, v(x), in this equation indicates that the relationship between real value and psychological value is nonlinear and instead is S-shaped. This dependence features three characteristics: dependence on reference, in which gains and losses are judged based on comparison with a reference point; loss aversion, in which the psychological effectiveness of a possible loss is larger than the equivalent gain; and diminishing marginal utility, in which the marginal utility is evaluated as a distance from the reference point (see Figure 1). The decision-making weight, π(p), is a subjective evaluation of probability and accords with the rule of “overestimation of small probability, underestimation of moderate and high probability” (Peng et al., 2019a).

Prospect theory can explain various phenomena related to crime. For instance, Van Gelder (2013) used the equation to demonstrate that the criminal population tends to have low subjective feelings regarding punishment but strong subjective feelings regarding rewards. In addition, this equation suggests that while escape from punishment after crime has a small probability, the criminal population overestimates this probability and thereby will risk danger in desperation (Mesch, 2000).

### Empirically based models and the commission of crime

Empirically based models have also been developed to explain criminal decision making (Gibson, 1981). Multiple researchers are using ethnographic methods to explore offenders’ criminal calculus while accounting for cultural and racial considerations, as available evidence suggests that crime rates vary among cultures, races, and demographics (Sampson and Lauritsen, 1997; Davies and Fagan, 2012). Investigators argue that improved comprehension of associations of criminal decision-making with recidivism rates can be gained by exploring how socioeconomic status of offenders is shaped and sustained by lifestyle characteristics (Esqueda et al., 2008; Espinoza et al., 2015). By using retrospective interviews to explore criminal decision making, research also point out that offenders’ efforts to earn the financial and social capital needed to cement, maintain, and reduce biopsychosocial stressors may generate a bounded rationality in which offenders discount or ignore punishment sensitivity and the formal risks of crime (Maschi et al., 2011; Richardson et al., 2020). Psychosocial risk factors have also been shown to affect decision-making and impulsivity, which may be associate with deviant or criminal behavior, such as drug or substance addiction, learning or intellectual disabilities, mental illness (e.g., bipolar I or II disorder), and ADHD (Gunnison and Mazerolle, 2007; Curcio et al., 2013; Aebi et al., 2014). Certainly, researchers have recognized that a model of criminal behavior also calls for knowledge about individual beliefs about legal threats and other sociocultural factors, such as cultural nuance, systemic failings, and oppressive systems, that directly impact the decision-making process (Riccio, 1992; Harris et al., 2011; Agovino et al., 2017).

In terms of specific cognitive processes, some researchers believe that differences in the decision-making process may cause individuals to continually perform non-adaptive behaviors, such as those linked to addiction (Redish et al., 2008). It was later proposed that crime may be related to such decision-making vulnerabilities or defects (Fried and Reppucci, 2001), and this view was validated by multiple studies in the fields of psychology and cognitive neuroscience (Luo et al., 2011). For example, Raine and Yang (2006) found that significant dysfunctions occur in the orbito-frontal lobe, cingulum gyrus, amygdala, thalamus, angular gyrus, and hippocampus of violent criminals. Importantly, the prefrontal lobe and amygdala are related to inhibitory control and emotional processing; they constitute the core cerebral areas of decision-making activities, and their dysfunctions may cause decision-making differences. The ventromedial prefrontal cortex is related to reward-punishment assessment and normal generation of emotions (Van Gelder and De Vries, 2014). According to dual-process theory, the results of experiments performed from a neurocognitive perspective indicated that functions of intuitive and emotive decision making were associated with weak to moderate correlations between neurocognitive measures that reflect localized activity in the prefrontal cortex and criminal behavior (Van Gelder, 2013). Accordingly, a series of studies found that damage in this region correlated related with crime, especially violent crime (Young et al., 2010; Chester et al., 2017). Luo et al. (2011) found that when various offenders performed the Iowa gambling task, they exhibited decision-making differences, such as insensitivity to punishment and the taking of blind risks, to different extents. Despite these and other advances, research on crime-related decision-making difference remains in its preliminary stages, and relatively few achievements have been reported (Van Gelder, 2013). Moreover, there has not yet been a systematic analysis performed regarding the probable associations of crime with different decision-making, and the limited research that has been performed has relied upon a small subset of research strategies, such as the Iowa gambling task (Bechara et al., 2005).

### Dual-process theory and the commission of crime

Another system that may provide insight into criminal decision-making is the dual-process theory, which holds that the decision-making process involves two processing systems: a heuristic system and an analytic system. The former involves intuitive and automated rapid processing, utilizes fewer psychological resources, and is significantly affected by emotion; the latter is a slow process based on cognitive efforts and consumes more cognitive resources (Brocas and Carrillo, 2014). Van Gelder (2013) believed that criminal decision-making results from the joint actions of analytic–heuristic processing systems. A criminal decision-maker not only logically analyzes potential profits (e.g., material profits and sexual satisfaction) and losses (e.g., sentence and damaged reputation) of crime as well as the probabilities of realizing these profits and losses, but it also is driven by emotion and intuition (moral shame, self-esteem, and pleasant sensation of crime). Subsequent research has begun to apply dual-process and has further proven the effectiveness of the dual-process model in crime prediction (Van Gelder and De Vries, 2014; Bitzer et al., 2016).

For example, researchers discovered that offenders that made decisions leading to criminal behaviors when the person was in circumstances that provoked great emotional arousal (heuristic system) tend to be less accountable to threats than offenders whose decisions were made when the potential criminal was less aroused. This difference occurs because high degrees of emotional arousal may eclipse thoughts of future consequences (analytic system) by absorbing all of the potential criminal’s attention on the present situation (De Neys and Glumicic, 2008; Van Gelder and De Vries, 2014; Poon, 2020). In particular, crime tends to carry immediate benefits driven by the heuristic system, whereas the costs of criminal behaviors tend to be more remote; therefore, the analytic system needs to be able to override the prepotent response of the heuristic system to prevent a crime from occurring (Hamilton, 2004; Meissner et al., 2005). Fried and Reppucci (2001) further confirmed that an offender may not be deterred by considerations of punishment severity and probability in the heuristic system, even though a person may be perfectly able to abstain from such acts in an emotionally neutral or analytic system.

The dual-process model suggests that some differences in the decision-making process may be related to crime. These differences include those related to a reference point, which can be defined as a subjective value selected and used by an individual for the purposes of comparison or appraisal of possible outcomes associated with a decision (Wang et al., 2015). In criminal behavior, the reference point is set too high, so that the individual feels highly dissatisfied with current living conditions (“All I want is to live better,” “This society is unfair. Rich people get rich through illegal ways, so why cannot I?”). Other such differences are deficient inhibitory control and impulsivity (“I did not think too much. I only wanted revenge.”), and shortsightedness and an unwillingness to strive to attain long-term goals, which suggest a weakness in delay satisfaction (“Work is too tiring and earning money legitimately is too slow; I want to get rich overnight.”). Finally, individuals with probability evaluation differences may underestimate the probability of punishment after committing a crime (“I felt the police would not find any evidence.”), they may adopt the decision-making style of shirking legal responsibility or ignoring ill feelings related to crimes (“It already happened. Do what you want.”), and they may calculate potential benefits and costs or weigh utilities differently (Bitzer et al., 2016).

### Current study

Based on the above analysis, we propose that heuristic and analytic functional differences may both be related to crime. First, in the heuristic system, the reference point may be set too high, leading to relative deprivation. Hence, we propose hypothesis 1: the decision-making styles of offenders tend to be more negative, and offenders are unwilling to think deeply; the relative deprivation due to the excessively high setting of a reference point relates to crime. Relative deprivation is the perception, by individuals or populations, of inferior living conditions. This perception comes after comparison of reality with the high reference, and it leads the experiencing of anger, dissatisfaction, or other negative emotions (Peng et al., 2021b). Reportedly, relative deprivation can significantly predict deviance, addiction, violence, and aggression, and it is one of the principal factors leading to crime (Dennison and Swisher, 2019).

Second, in the analytic system, the sensitivity to reward–punishment may be different. We, therefore, propose hypothesis 2: offenders are more shortsighted, and they possess weaker delayed satisfaction ability. Crime may be associated with an excessively high sensitivity to reward and low sensitivity to punishment; such individuals would overestimate the profits from crime and underestimate the losses and consequently believe that crime is a cost-efficient decision-making behavior. According to conclusions drawn from previous studies on the dual-process theory as applied to criminal behavior, offenders prefer to incur immediate benefits, which is provoked by the heuristic system (De Neys and Glumicic, 2008; Poon, 2020); in other words, offenders may make decisions without careful consideration (analytic system), which is manifested as irrationality and the lack of long-term considerations (Hamilton, 2004; Malesza and Ostaszewski, 2020). Experimentally, as previously described, a lack of long-term thinking can be evaluated using delay discounting tasks (Reynolds and Schiffbauer, 2004), and irrationality can be assessed using questionnaires that investigate decision-making styles.

In this study, then, based on the dual-process model, offenders and non-offenders among adults in a Chinese population were compared in terms of decision tasks and scales to assess which decision-making differences were related to crime.

## Materials and methods

### Sample

For the purpose of comparing the difference of decision-making between offenders and non-offenders people, convenience sampling was used in the distribution of questionnaires to several prisons, logistics and architecture companies, community centers and colleges in Sichuan and Shandong provinces of China between December 2020 and April 2021. The data were initially collected from prisons in order to perform preliminary statistical analyses and get the demographic characteristics of offenders. When recruit non-offenders, efforts were made to match their demographic variables, such as gender, age and educational qualifications, with those of the offending group. For example, in the logistics and architecture company, we firstly got the staff list and their demographic information from the personnel department. Then we can designedly to recruit the non-offenders to make the demographic compositions similar with the offending group. Additionally, each participant of the non-offending group was screened by answering if he/she has any criminal records. A total of 1,231 paper-pencil questionnaires were administered in the reading rooms, and ultimately 1,154 valid questionnaires were returned, for a response rate of 93.74%. All participants provided written informed consent before completing the tests, and they were awarded 10 RMB (approximately 1.8 USD). The research described in this paper meets the ethical guidelines of the author’s University and has been approved by its ethics committee.

The sample population consisted of 518 offenders (44.89%) and 636 non-offenders young adults (55.11%). The participants ranged in age from 18 to 24 years (mean, 21.85 years; SD, 3.18 years), and all were unmarried. The mean ages of the offending group and the non-offending group were 21.34 years and 22.04 years, respectively. The two groups were not significantly different in gender (χ^2^ = 1.49, *P* = 0.22), education level (χ^2^ = 4.07, *P* = 0.25), registered residence (urban or rural, χ^2^ = 0.11, *P* = 0.75), or ratio of status as a single child to non-single child (χ^2^ = 2.66, *P* = 0.11) (Table 1).

**TABLE 1:** Sample compositions between offending or non-offending groups (*n* = 1,154).

		Offending group	Non-offending group
Average age		21.34	22.04
Gender	Male	386 (74.52%)	452 (71.07%)
	Female	132 (25.48%)	184 (28.93%)
Registered residence	Urban	164 (31.66%)	207 (31.60%)
	Rural	354 (68.34%)	429 (68.40%)
Status of only child	Yes	201 (38.80%)	277 (43.55%)
	No	317 (61.20%)	359 (56.45%)
Education level	Elementary school and below	67 (12.93%)	72 (11.32%)
	Middle school	242 (46.72%)	307 (48.27%)
	High school	178 (34.36%)	202 (31.76%)
	Junior college and above	31 (5.99%)	55 (8.45%)

### Measurements

#### Personal relative deprivation scale

An adapted version of PRDS, which was originally developed by Callan et al. (2011), was used in this study. Notably, Peng et al. (2019a) translated PRDS into Chinese and found that some items in the original PRDS are unfit for use with Chinese people. The Chinese version of PRDS involves a total of three items; an example item is “I feel deprived when I think about what I have compared to what other people like me have.” Responses were made on a 6-point scale, ranging from 1 (strongly disagree) to 6 (strongly agree) (Callan et al., 2011). The extent to which people’s general perceptions and emotions relate to those of others with similar demographic characteristics is indicated by the total PRDS score, which is calculated as the sum of all the item scores, with two items being reverse scored. A higher total score means stronger relative deprivation and reflects a higher setting level of the reference point; in other words, a subject with a higher total score is more unsatisfied with the current situation. The internal consistency coefficient of the scale as applied in the current study was 0.89.

#### Sensitivity to punishment and sensitivity to reward questionnaire

Sensitivity to punishment and sensitivity to reward questionnaire (SPSRQ), developed by Torrubia et al. (2001), was translated into Chinese and revised by Li et al. (2007). The Chinese version of SPSRQ consists of two subscales (sensitivity to reward and sensitivity to punishment). Examples items are: “When you were still a kid, were you worried about punishment by your family members or from schools?” (punishment), and “Do you often do things to be praised?” (reward). Each item was answered with either “yes” (score of 1) or “no” (score of 0). The calculation of each subscale score is the sum of the scores of the corresponding items, and a larger subscale score indicates a higher sensitivity to reward or punishment. The internal consistency coefficients of the sensitivities to reward and punishment were 0.70 and 0.78, respectively.

#### Monetary choice questionnaire

Monetary choice questionnaire (MCQ) was used in this study to evaluate delay discounting. This scale has 27 items. For each item, participants were asked to choose between a smaller immediate profit and a larger long-term profit (e.g., “Would you choose $50 in the future 3 weeks or $27 at present?”). The delay discounting rate, K, of each individual was calculated with the equation K = A/(1 + VD). Since K values typically obey a non-normal distribution, the score of this scale was expressed as log(K). A larger log(K) means the subject tends to choose a short-term reward and thus is more shortsighted and impulsive, which reflects weaker delay of satisfaction ability (Peng et al., 2019b). The internal consistency coefficient of MCQ in this study was 0.74.

#### Melbourne decision-making questionnaire

Melbourne decision-making questionnaire (MDMQ), developed by Mann et al. (1997) was used in this study to evaluate decision-making styles. Peng et al. (2021a) translated MDMQ into Chinese and revised the scale. The Chinese version of MDMQ consists of three subscales (vigilance, buck-passing, and hesitation) and a total of 19 items. Responses were made on a 3-point scale, ranging from 1 (disagree) to 3 (agree). Adaptive or competent decision-making styles were indicated by higher scores on the vigilance subscale, which included questions such as “I like to consider all of the alternatives when I make a decision.” Avoidant styles were characterized by higher scores on the buck-passing subscale, which included questions such as “I prefer to leave decisions to others.” Indecisive and defective decision-making style were indicated by higher hesitation scores, on questions such as “I cannot think straight if I have to make a decision in a hurry.” The scores of all items from each subscale were added and used as the subscale score. Specifically, vigilance was considered a positive and rational decision-making style, while buck-passing and hesitation were considered negative decision-making styles (Peng et al., 2021a). The internal consistency coefficients of the three subscales fell within the range of 0.79–0.87.

### Data analysis

For the purposes of exploring the decision-making difference between offenders and non-offenders, the following analytic plan was adopted. First, the differences of decision-making in the context of the current study (such as decisions related to relative deprivation, sensitivity to reward–punishment, and the delay discounting rate) were compared between adult offenders and non-offenders. Second, a correlation analysis was conducted to determine whether decision-making differences were significantly correlated. Finally, with the type of participants (offenders or non-offenders) as the dependent variable, logistics regression analyses were performed with relative deprivation, sensitivity to reward–punishment, delay discounting rate, and decision-making style as independent variables. All data analyses were conducted with Statistical Package for the Social Sciences for Windows (version 19).

## Results

### Comparison of decision-making differences between groups

This study firstly compared differences in decision-making between groups of offenders and non-offenders. The results (Table 2) indicated that compared with non-offenders, offenders have higher relative deprivation (*t* = 6.82, *p* < 0.01), sensitivity to reward (*t* = 5.12, *p* < 0.01) and delay discounting rate (*t* = –6.55, *p* < 0.01), but they have lower sensitivity to punishment (*t* = –6.55, *p* < 0.01) and vigilance (*t* = –8.68, *p* < 0.01). The two groups were not significantly different with regard to buck-passing (*t* = 1.61, *p* = 0.11) or hesitation (*t* = 1.55, *p* = 0.12).

**TABLE 2:** Decision-making differences between offenders and non-offenders (*n* = 1,154, Mean ± SD).

	n	Relative deprivation	Sensitivity to reward	Sensitivity to punishment	Delay discounting rate	Vigilance	Buck-passing	Hesitation
Offenders	518	8.55 ± 3.64	9.33 ± 3.98	6.77 ± 3.03	−1.22 ± 1.47	13.62 ± 2.28	7.44 ± 1.84	17.01 ± 3.96
Non−offenders	636	7.13 ± 3.38	8.14 ± 3.85	8.00 ± 3.33	−1.82 ± 1.33	14.56 ± 2.22	7.24 ± 1.80	16.65 ± 4.05
*T*		6.82	5.12	−6.55	7.18	−8.68	1.61	1.55
*P*		<0.01	<0.01	< 0.01	<0.01	<0.01	0.11	0.12

### Logistic regression analysis of the decision-making differences on offending

Correlation analysis showed that delay discounting rate, decision-making styles, sensitivity to the reward–punishment continuum, and relative deprivation correlated significantly (*p* < 0.01). To further investigate decision-making differences as risk factors for offending, the type of participants (offending or non-offending) was adopted as the dependent variable. Binary logistics regression analyses were conducted with independent variables including relative deprivation, sensitivity to reward–punishment, delay discounting rate, and decision-making styles. The results of these analyses demonstrated that relative deprivation, sensitivity to reward, and delay discounting rate were all significantly positively associated with the likelihood of offending, but that sensitivity to punishment and vigilance were all significantly negatively associated with the likelihood of offending. However, neither buck-passing nor hesitation was significantly associated with offending. Analysis of the Wals value, which indicates the contribution of an independent variable to the prediction of the dependent variable, showed that sensitivity to punishment most severely impacted the offending (Table 3).

**TABLE 3:** Logistics regression analysis of decision-making differences against offending (*n* = 1,154).

Independent variable	B	S.E,	Wals	Sig.	Exp (B)	Exp (B) 95% confidence interval
Relative deprivation	0.07	0.02	12.46	<0.01	1.07	1.03–1.11
Sensitivity to reward	0.04	0.02	6.31	0.01	1.05	1.01–1.08
Sensitivity to punishment	−0.12	0.02	26.85	<0.01	0.89	0.85–0.93
Delay discounting rate	0.18	0.05	13.20	<0.01	1.20	1.09–1.32
Vigilance	−0.68	0.18	13.94	<0.01	0.51	0.35–0.72
Buck-passing	0.10	0.16	0.39	0.53	1.11	0.81–1.51
Hesitation	0.08	0.16	0.22	0.64	1.08	0.78–1.49

## Discussion

Based on the dual-process model, the current study was designed to identify decision-making differences between offenders and non-offenders. For this purpose, we compared the differences between offenders and non-offenders in terms of reference point setting, sensitivity to the reward–punishment continuum, delay discounting rate, and decision-making style. Moreover, the correlations among the above variables and their contributions to the prediction of crime were analyzed. The current survey-based study reveals several interesting findings regarding the decision-making patterns of offenders, and our findings are of significance to the strengthening of education and rehabilitation of offenders in the pursuit of crime reduction.

First, this study indicated that emotions play an important role in the decision-making of offenders, which was driven by the heuristic system. The current study showed that, compared with the reference individuals, offenders had higher reference points and thus experienced more relative deprivation. Relative deprivation is not only a perception about a person’s socioeconomic conditions, but it also involves discontent and indignation toward other individuals and toward society (Peng et al., 2021b). People with high relative deprivation tend to have a strong desire to leave behind perceived inferior living conditions, and the emotional components of relative deprivation (e.g., fury, frustration, and apathy) make individuals irritable and impulsive, and drive them emotionally to make irrational decisions (Burraston et al., 2018). According to the dual-process view of criminal decision making, emotional arousal of the heuristic system harnesses the criminal’s decision-making process and does so in ways that are difficult to reconcile with a rational analytic system (Cornish and Clarke, 1989). In addition, higher reference points lead to interpersonal hostility and further causes a lowering social adaptability, lagged moral development, and aggressiveness, all of which are related to crime (Greitemeyer and Sagioglou, 2017).

Second, this study also indicated that the analytic system of the decision process for offenders is inaccurate or at least incomplete. As compared with the reference group, offenders were found to have a higher sensitivity to reward and a lower sensitivity to punishment, and they were more likely to pursue excitement and profits while ignoring or underestimating risks and losses. These qualities suggest that offenders are willing to risk danger for the sake of even a trifling profit (Shulman and Cauffman, 2013). Third, offenders were found to have a weaker delayed satisfaction ability; they tended to pursue instant rewards, to be shortsighted and impulsive, and to be unwilling to strive to attain long-term goals (Lee et al., 2017). Fourth, in terms of decision-making styles, offenders and non-offenders were significantly different with regard to the dimension of vigilance. The decision-making of offenders was more dependent on intuition, and they were unwilling to search for more information with which to make a decision or to compare the potential successes and failures or pros and cons of alternative schemes. In other words, our results suggest that offenders usually make hasty and low-quality decisions.

Based on the results of this study, the decision-making patterns of offenders can be summarized as follows. Offenders are often dissatisfied with their current socioeconomic conditions, and they believe that such inferior living conditions are caused by social unfairness, so they have a strong motivation to escape such inferior living conditions, which will cause a range of negative emotions that drive the heuristic system. Additionally, the inaccurate analytic systems of offenders overestimate the cost-benefit ratio that is posed by the threat of legal punishment, and they underestimate the negative effects of crime on society. In summary, the decision-making of offenders is hasty and shortsighted and is full of emotionalism, and their abilities to delay satisfaction are weaker as compared to those of non-offenders.

### Implications of the findings

Providing insight into efforts toward the prevention of crime, the current study reveals some risk factors associated with the criminal decision-making process. For the purposes of the prevention of crime, the results of current study indicated that attention should focus on juveniles with low sensitivity to punishment, and efforts should be made to cultivate reverence for the law. In addition, technology and methodologies should be adapted to strengthen the cultivation of rational decision-making, including the development of positive decision-making styles and the ability to select optimal options. These processes have been found to be important individual dispositions that are predictive of criminal behavior (McClanahan et al., 2019). Social organizations and lawmakers also should respond to developmental differences between people in formulating crime policies, and the rule of law should be strengthened in a manner that promotes social fairness, which is a crucial contextual factor that has been shown to directly correlate with crime rate (Bloch, 2011).

This study additionally provided enlightenment for the theoretical and practical study of crime prevention and criminal education. We investigated differences in indices, including relative deprivation, leading to several notable achievements. Criminal decision-making is one step before the enactment of criminal behavior, and it is thus the cognitive process that is the closest to criminal behavior (Van Gelder, 2013). If we can identify which irrational vulnerabilities in the decision-making process are related to crime, we can design techniques to mitigate these vulnerabilities and thus inhibit crime-related behaviors. Identification of relevant vulnerabilities can also play a role in research into the reconstruction of criminal decision-making and the education of offenders. For instance, we found that low delay discounting and impulse are associated with crime, so methods that improve inhibitory control, such as task-switching training and neural feedback training, may be emphasized in the education of offenders (Kiesel et al., 2010).

### Limitations

Although this study led to meaningful findings, the limitations of this study also need to be discussed. First, the sample and survey method of this study may affect the ability to generalize the research conclusions. The drawing of conclusions about a Chinese population using the non-random sampling method may have led to sample representativeness bias. For example, age is an important influencing factor to crimes. However, all of our participants were aged from 18 to 24, falling to make comparison between these young adults to older adults. Juvenile is a crucial stage of development marked by rapid changes in physical, cognitive, social, and affective development, and Juveniles lack self-control and are less culpable for their offending behavior (Farrington, 1986; Mann et al., 1989; Steinberg and Scott, 2003). So the participants of current study were without broad representation in terms of age. Additionally, when recruit non-offenders adults, efforts were made to match their demographic variables, such as gender, age, registered residence and educational qualifications, with those of the offending group. In this way, we make the demographic compositions of non-offenders similar with the offending group and just focused on the decision making differences between the two groups. However, strictly speaking, the so-called non-offending group is not a good sample of ordinary young adults that have no history of delinquency. In general, the current study ignored the important impacts of demographic variables on offending.

Another important issue is that there may be offenders in the non-offending group. Though we used a screening question as to whether they have committed a crime, some participants may disguise his/her criminal record. Additionally, the non-offending group could also include those who have committed crimes, but just have not been caught.

Third, the current study adopted the measurement of relative deprivation in an offenders sample and compared it to that of a public sample. This strategy may have affected the accuracy of the measurement of relative deprivation because it is expected that offenders would score higher on measures of relative deprivation, as they are currently deprived by virtue of their punishment.

### Future research directions

First, future studies are encouraged to sample more representatively. For example, older offenders can be included. Additionally, more attention should be paid on the truly existed demographic differences between offenders and non-offenders, and the correlational relationship of these demographic differences with decision making differences leading to crimes. Second, in the current study, we regarded offenders as a whole. However, difference types of crimes may be related with different decision making components. So it is valuable to explore and identify the dominant decision making differences related with a particular kind of crime. Third, more decision making differences should be considered. For example, crime may also be associated with differential perception of risk or feedback learning (Esqueda et al., 2008), and pursuit of these additional factors warrants additional research in future studies. Last, longitudinal studies that can reveal the causal relationship of decision making differences and crime are especially welcome.

## Conclusion

The current study explored the decision-making differences of offenders and non-offenders based on the dual-process model in order to shed light on criminal behavior. Whereas traditional frameworks and models assume the criminal act to be the result of cognitive assessment, our results indicated that crime may originate both from the driving of emotion in the heuristic system and the inaccurate or at least incomplete cognitive appraisals in the analytic system. The findings of the current study have interesting implications for efforts to reduce criminal behaviors. By comprehending how individual differences drive decision-making, and how the decision-making process may in turn increase or decrease criminal behavior in different social contexts, we cannot only strengthen theory but also improve interventions. Specifically from the results of this study, efforts should be taken to appeal both to the deliberative system and to the intuitive system to reduce criminal behavior.

## Data availability statement

The raw data supporting the conclusions of this article will be made available by the authors, without undue reservation.

## Ethics statement

The studies involving human participants were reviewed and approved by the Committee on Human Experimentation at the Chengdu University. The patients/participants provided their written informed consent to participate in this study.

## Author contributions

JP, PF, and JT designed the study, performed data collection, and analyzed the data statistically. JP, JZ, WY, XZ, JT, and PF wrote the manuscript. All authors read and approved the final manuscript.
